# Evaluation of the impact of calorie labeling on McDonald’s restaurant menus: a natural experiment

**DOI:** 10.1186/s12966-019-0865-7

**Published:** 2019-11-04

**Authors:** Joshua Petimar, Maricelle Ramirez, Sheryl L. Rifas-Shiman, Stephanie Linakis, Jewel Mullen, Christina A. Roberto, Jason P. Block

**Affiliations:** 1 Department of Epidemiology, Harvard T.H. Chan School of Public Health, 677 Huntington Avenue, Boston, MA 02115 USA; 2 Division of Chronic Disease Research Across the Lifecourse, Department of Population Medicine, Harvard Medical School and Harvard Pilgrim Health Care Institute, Boston, MA USA; 3 Brigham and Women’s Hospital, Boston, MA USA; 4 Departments of Population Health and Internal Medicine, The University of Texas at Austin Dell Medical School, Austin, TX USA; 5 Department of Medical Ethics and Health Policy, Perelman School of Medicine, University of Pennsylvania, Philadelphia, PA USA

**Keywords:** Calorie labeling, Menu labeling, Nutrition policy, Natural experiment, Obesity prevention

## Abstract

**Background:**

The long-term effect of calorie labeling on fast-food purchases is unclear. McDonald’s voluntarily labeled its menus with calories in 2012, providing an opportunity to evaluate this initiative on purchases.

**Methods:**

From 2010 to 2014, we collected receipts from and administered questionnaires to 2971 adults, 2164 adolescents, and 447 parents/guardians of school-age children during repeated visits to 82 restaurants, including McDonald’s and five control chains that did not label menus over the study period in four New England cities. In 2018, we analyzed the data by using difference-in-differences analyses to estimate associations of calorie labeling with calories purchased (actual and estimated) and predicted probability of noticing calorie information on menus.

**Results:**

Calorie labeling at McDonald’s was not associated with changes in calories purchased in adults (change = − 19 cal pre- vs. post-labeling at McDonald’s compared to control chains, 95% CI: − 112, 75), adolescents (change = − 49 cal, 95% CI: − 136, 38), or children (change = 13 cal, 95% CI: − 108, 135). Calorie labeling generally increased the predicted probability of noticing calorie information, but did not improve estimation of calories purchased.

**Conclusions:**

Calorie labeling at McDonald’s was not associated with changes in calories purchased in adults, adolescents, or children. Although participants were more likely to notice calories on menus post-labeling, there was no improvement in ability to accurately estimate calories purchased.

## Background

In May 2018, restaurant chains with 20 or more locations in the United States were mandated to label their menus with calorie information to comply with the menu labeling provision of the 2010 Patient Protection and Affordable Care Act (ACA) [1]. Policymakers adopted this requirement to increase awareness of the calorie content of prepared food purchased outside the home, especially restaurant food, where calories are underestimated by restaurant patrons [2–4]. The federal policy also preempted city and state laws requiring calorie labeling, establishing uniform requirements for chain food establishments across the country [5]. The downstream goal of the law is to enhance diet quality by changing consumer behavior and encouraging food retailers to offer lower calorie items. In part, due to several delays in implementation of the law [5], many large restaurant chains began voluntarily posting calories on their menus before it was required, including McDonald’s, which began labeling in September 2012.

Despite the popularity of calorie labeling [6] and the federal requirement, the effectiveness of this policy for reducing calories purchased in restaurants is unclear. Although some previous observational and experimental studies have found that calorie labeling reduces calories purchased [7–11], other studies have found no difference, especially those conducted in real-world settings, primarily at fast-food restaurants [12–19]. Importantly, many studies that previously examined this association lacked appropriate comparison groups [20]. Few studies in adolescents and children have had large enough samples to examine this association overall or in subgroups [14, 17, 21].

To address these gaps, we conducted a study to evaluate McDonald’s calorie labeling on customers’ actual and estimated calorie content of purchased foods, compared to customers of five fast-food chains that did not implement labeling over the study period. We examined this separately in adults, adolescents, and children.

## Methods

### Study area and restaurant selection

We capitalized on a natural experiment to examine calorie purchases before and after voluntary calorie labeling at McDonald’s in 2012 compared to a group of control restaurants: Burger King, Subway, KFC (except for adolescents), Wendy’s, and Dunkin Donuts (only for adolescents). These were chosen as control restaurants because they are similar to McDonald’s in popularity, price point, and types of meals served. Further, all have a wide range of menu offerings in terms of calories, allowing us to detect changes in overall trends in calorie purchases independent of labeling. Other details regarding the process and rationale for restaurant selection have been described previously [2]. Our study area included Boston and Springfield, Massachusetts; Hartford, Connecticut; and Providence, Rhode Island, four large New England cities with substantial racial and socioeconomic diversity. For adults and children, we randomly selected three McDonald’s, three Burger Kings, two Subways, one KFC, and one Wendy’s in each city in 2010. These restaurant chains were chosen because they had at least two locations in each city and offered dinnertime meals. Restaurants that had closed or whose management refused participation in 2010 were replaced by randomly selecting another restaurant of the same chain in the same city. We revisited the same restaurants every year from 2011 to 2014 except when management refused to participate, in which case we selected the nearest restaurant of the same chain. We sampled adults and children in 48 restaurants, 37 of which were included in both the pre-intervention (i.e. 2010–2012) and post-intervention periods (i.e. 2013–2014). Data collection in 2012 was limited and only done to supplement collection in restaurants that were added in 2011. We collected participant surveys and receipts in the evenings from April to August in each year of data collection.

The restaurant selection procedure was similar for adolescent participants, except we chose restaurant chains with at least two sites within one mile of a high school (three McDonald’s, two Burger Kings, two Subways, two Dunkin Donuts, and one Wendy’s in each city) and enrolled participants in the early afternoon during the school year and at lunchtime over the summer. We believed this sampling strategy would help us recruit adolescents, especially after school when they may be unaccompanied by adults, thereby minimizing the influence of parent and guardian preferences. We excluded restaurants poorly attended by adolescents; this resulted in more exclusions in the pre-intervention period for adolescents (*n* = 11) than we had for adults and children (*n* = 5). As a result, we visited more restaurants to recruit the adolescent sample than we did for adults and children. Overall, we sampled adolescents from 53 restaurants, 37 of which were included in both the pre- and post-intervention periods. We collected surveys from June to August in each year of data collection; in Boston only, we collected a separate after-school sample from April through June in each year of data collection. This study was approved by the institutional review board of Harvard Pilgrim Health Care.

### Participant enrollment

We invited all adults (≥18 years), adolescents (11–20 years), and parents or legal guardians of children (3–15 years) to participate. While there was an overlap in age eligibility for the groups, we enrolled them at different times of day, making it unlikely that any participants were included in both samples. As described previously [2], we approached restaurant customers as they entered the restaurant and requested they return the receipt and complete a questionnaire upon exiting in exchange for a $2 incentive. After participants returned their receipt, we administered a questionnaire asking them to identify which items on the receipt were purchased for their personal consumption (or their child’s for the children sample). With the questionnaire, we further assessed details that were not clear from the receipt, such as whether items were shared, the use of sauces/condiments, the addition of cheese, the type of salad dressing, and specific beverage choices. We also asked participants to estimate their meal’s calorie content and assessed participant characteristics. We gathered all information directly from adults and adolescents; for children, all questions were directed to their parent or legal guardian. Although adults and children were enrolled at the same restaurants and times, we did not include parents or legal guardians in the adult sample if their accompanying child was enrolled (we preferentially enrolled children when an adult was accompanying a child). We administered questionnaires in English, but a Spanish language version of the recruitment script was available to facilitate recruitment of Spanish speakers. The overall response rate was approximately 42% for adults (40% pre-intervention and 45% post-intervention), 46% for adolescents (43% pre-intervention and 51% post-intervention), and 44% for children (44% both pre- and post-intervention).

### Outcomes

Our primary outcome was total calories purchased for each participant, which was calculated by linking items purchased for participants’ consumption to nutrition information from restaurant websites (collected in July of each year of the study) and summing the total calories purchased for each participant. Estimated total calories purchased was a secondary outcome because we wanted to determine if labeling helped consumers understand the overall calorie content of their meals (even if they did not purchase fewer calories). We also included whether participants noticed calorie information on menus as a secondary outcome. Both of these were assessed on questionnaires.

### Covariates

We measured participant characteristics that we hypothesized might affect response to labeling on questionnaires, including age, sex, race/ethnicity (“White,” “Black,” “Hispanic,” “Asian” and/or “Other”), and self-reported weight and height, which we used to calculate body mass index (BMI). We also asked participants how important price, taste, convenience, and the total number of calories were when deciding which items to order at the restaurant (“not at all”, “a little”, or “a lot”).

### Statistical analysis

We conducted analyses separately in adults, adolescents, and children. We excluded participants whose estimated or actual calorie intake exceeded 5000 cal (< 1% in all samples) and those who had missing data on any of the covariates in our main model (*n* = 179 adults [6%], *n* = 115 adolescents [5%], *n* = 72 children [14%]).

For our primary analyses examining the association between calorie labeling and calories purchased after labeling in 2012, we fit multivariable generalized estimating equations (GEE) that included indicator variables for group (McDonald’s vs. other) and period (pre- vs. post-labeling), an interaction term between group and period (β_interaction_), which estimated the effect of calorie labeling, and covariates whose distributions appeared to change slightly over time differently between groups: age, sex, race/ethnicity, BMI, city, and restaurant chain. In adolescents and children, we adjusted for BMI-for-age-and-sex z-score, calculated from national reference data [22], instead of BMI. We included these covariates because if the population composition of the two intervention groups changed differently over time, and were related to calorie purchases, this could bias the association between labeling and calorie purchases. To account for correlation between purchases in the same restaurant, we included restaurant location as a random effect. We additionally examined differences in calories purchased between the post- and pre-intervention periods within each intervention group.

In secondary analyses, we examined differences in the predicted probability of underestimating the calorie content of purchased meals, as well as whether participants noticed calorie information on menus. For each analysis, we excluded individuals missing data on the respective outcome (across samples: 7–12% missing calorie underestimation; < 1–2% missing noticing calorie information). We ran multivariable GEEs, adjusting for the same covariates as in our primary analysis, and obtained standardized predicted probabilities of each outcome [23, 24] within each group and period. We then found the difference in mean standardized predicted probabilities between the post- and pre-intervention periods for each intervention group, calculated the difference-in-differences, and obtained 95% confidence intervals (CI) from 1000 bootstrapped samples. In sensitivity analyses, we considered underestimation of calories purchased as a continuous measure, rather than a binary measure. We additionally calculated the proportion of customers who reported using calorie labels to make purchasing decisions among those who said they noticed the calorie labels in McDonald’s in the post-labeling period.

One important assumption of difference-in-differences analyses is that the pre-trend values for the outcomes are similar in the intervention and control groups. To test this, we ran the primary models described above with observations from 2010 and 2011 only and evaluated the interactions between intervention group and time (there were too few participants enrolled in 2012 to include in this analysis). We did not detect any significant interactions (P-interaction > 0.20 for all), and therefore had no evidence that the pre-intervention trends in the outcomes differed between intervention and control groups.

We conducted sensitivity analyses where we repeated all primary and secondary analyses additionally adjusting for importance of calories, convenience, price, and taste in participant food choices, as well as whether participants properly estimated recommended daily calorie intake. We also reexamined associations between calorie labeling and calories purchased after excluding McDonald’s customers who did not report seeing calorie labeling after implementation. Lastly, we conducted exploratory subgroup analyses in which we repeated our primary analysis within strata of participant sex (male/female), weight status (obesity/no obesity), and race/ethnicity (Black/Hispanic/White); we could not explore these in purchases made for children due to low sample size.

All statistical analyses were conducted in SAS version 9.4 (Cary, NC). We calculated 2-sided 95% CIs for all statistical tests.

## Results

The study population after exclusions (Table 1) included 2971 adults (31% McDonald’s customers; mean age, 37.6 years [SD, 15.9]; 43% female), 2164 adolescents (41% McDonald’s customers; mean age, 16.3 years [2.7]; 48% female), and 447 children (41% McDonald’s customers; mean age, 8.2 years [3.1]; 51% female). Most participants were non-White (60 to 84%) across all samples. Among adults, compared to control chains, McDonald’s customers were more likely to be female and Black; school-age children at McDonald’s were also more likely to be Black. Among adolescents, a higher proportion of McDonald’s customers were in Boston compared to the control restaurants due to the additional sample during the school year. The mean calories purchased was 807 (range: 0–4000) for adults, 746 (range: 0–2980) for adolescents, and 694 (range: 90–2170) for children. About half of participants across age groups responded that calories influenced their meal selection to some degree, and most answered that that recommended daily calorie intake was between 1000 and 3000 cal.

**Table 1:** Characteristics^a^ of Participating Adults, Adolescents, and Children by Restaurant Chain and Timing of Purchase

	Adults				Adolescents				Children			
	McDonald’s		Other chain		McDonald’s		Other chain		McDonald’s		Other chain	
	Pre (*n* = 579)	Post (*n* = 343)	Pre (*n* = 1294)	Post (*n* = 755)	Pre (*n* = 546)	Post (*n* = 332)	Pre (*n* = 752)	Post (*n* = 534)	Pre (*n* = 126)	Post (*n* = 59)	Pre (*n* = 177)	Post (*n* = 85)
Age, years	37.9 (16.7)	38.3 (15.7)	36.9 (15.9)	38.1 (15.2)	15.8 (2.9)	15.7 (2.7)	16.6 (2.7)	16.7 (2.5)	7.8 (3.0)	8.5 (3.1)	8.4 (3.3)	8.2 (3.1)
BMI, kg/m^2^	28.0 (6.1)	27.6 (5.8)	27.9 (6.2)	28.1 (6.0)	23.1 (4.5)	23.5 (5.0)	23.6 (4.8)	24.2 (5.3)	20.0 (6.0)	22.1 (7.0)	22.2 (6.9)	22.3 (7.6)
BMI-for-age-and-sex z score	–	–	–	–	0.52 (0.92)	0.54 (1.09)	0.49 (1.03)	0.59 (0.98)	0.76 (1.91)	1.11 (1.67)	1.24 (1.54)	1.27 (1.69)
Sex (%)												
Male	52	52	60	61	51	55	52	49	50	51	49	48
Female	48	48	40	39	49	45	48	51	50	49	51	52
Race/ethnicity (%)												
White	34	38	40	40	17	17	22	20	15	17	24	15
Black	34	36	29	29	32	35	34	38	37	36	27	33
Hispanic	20	17	19	20	30	25	26	25	28	29	33	35
Asian	4	2	4	5	8	8	5	5	3	3	3	4
Other	9	8	9	7	14	14	13	11	17	15	12	13
City (%)												
Boston	28	39	24	37	55	70	44	54	29	44	35	52
Hartford	28	21	34	24	16	16	17	14	33	19	35	21
Providence	30	28	22	27	18	7	25	20	23	19	16	15
Springfield	14	12	20	11	11	7	14	12	15	19	14	12
Restaurant (%)												
McDonald’s	100	100	–	–	100	100	–	–	100	100	–	–
Burger King	–	–	43	48	–	–	35	29	–	–	54	67
Kentucky Fried Chicken	–	–	17	16	–	–	–	–	–	–	20	12
Subway	–	–	25	22	–	–	21	21	–	–	14	14
Wendy’s	–	–	15	14	–	–	16	18	–	–	12	7
Dunkin’ Donuts			–	–	–	–	28	32	–	–	–	–
Importance of calories in selecting meals (%)												
Not at all	49	47	49	50	46	47	51	55	48	37	40	34
A little	26	26	25	22	32	31	30	27	23	27	30	27
A lot	24	27	26	28	23	22	18	19	30	36	30	39
Estimation of daily calorie needs (%)												
< 1000	20	26	17	20	27	26	19	21	35	46	28	44
≥ 1000- < 2000	40	45	45	45	33	37	37	41	41	34	43	35
≥ 2000- < 3000	33	23	30	26	31	25	34	28	17	16	23	18
≥ 3000	8	6	8	8	8	12	10	9	6	4	6	3

^a^Mean (standard deviation) or %

In multivariable-adjusted models, the calorie content of meals declined by 80 cal in McDonald’s (95% CI: − 155, − 4) and by 60 cal in control restaurants (95% CI: − 116, − 5) in the post- compared to the pre-intervention period among adults. In difference-in-differences models comparing McDonald’s to control restaurants before vs. after labeling, there was no change in calories purchased associated with calorie labeling (β_interaction_ = − 19 cal, 95% CI: − 112, 75) (Table 2). We similarly observed decreases in calories purchased among children in both McDonald’s and control restaurants, but again no change in calories purchased associated with labeling (β_interaction_ = 13 cal, 95% CI: − 108, 135). In adolescents, we observed neither a decline in calories purchased over time nor a change associated with labeling; the β_interaction_ was the largest for this group though (− 49, 95% CI -136, 38). Results were similar when adjusting for additional covariates in sensitivity analyses (Additional file 1: Table S1) and when restricting the analysis to McDonald’s customers who reported seeing calorie information on menus (Additional file 1: Table S2).

**Table 2:** Multivariable-Adjusted Changes (95% CI) in Calories Purchased by Adults, Adolescents, and Children After Calorie Labeling in McDonald’s Restaurants Compared to Other Fast-Food Restaurants

	McDonald’s				Other Chains				Difference-in-differences (adjusted)^b^
	Pre^a^	Post^a^	Change (crude)	Change (adjusted)^b^	Pre^a^	Post^a^	Change (crude)	Change (adjusted)^b^	
Adults	718 (474)	630 (425)	−89 (− 168, −10)	−80 (−155, −4)	888 (455)	816 (468)	−72 (−127, −16)	−60 (−116, −5)	−19 (−112, 75)
Adolescents^c,d^	759 (488)	709 (408)	−51 (− 123, 21)	−40 (− 106, 26)	756 (427)	739 (459)	−17 (−82, 48)	1 (− 48, 50)	−49 (− 136, 38)
Children^d^	719 (369)	585 (280)	− 134 (− 214, − 53)	− 158 (− 231, −86)	766 (345)	585 (345)	− 180 (−291, − 69)	− 159 (− 257, − 61)	13 (− 108, 135)

^a^Mean (SD) presented

^b^Adjusted for age (years, continuous), sex (female [ref], male), race/ethnicity (white [ref], black, Asian, Hispanic, other), BMI (kg/m^2^, continuous), city (Boston [ref], Hartford, Providence, Springfield), and restaurant chain (Burger King [ref], KFC, Subway, Wendy’s). Restaurant location was included as a random effect

^c^Control restaurants included Burger King (ref), Wendy’s, Subway, and Dunkin’ Donuts

^d^Adjusted for BMI-for-age-and-sex-z score (continuous) instead of BMI

We did not observe any changes in predicted probability of underestimating calories purchased in either McDonald’s (predicted change = 1, 95% CI: − 6, 8) or control restaurants (predicted change = − 1, 95% CI: − 5, 4) in the post- compared to the pre-intervention period among adults, and, in differences-in-differences models, there was no association of calorie labeling on ability to accurately estimate calories purchased (β_interaction_ = 1, 95% CI: -7, 9). Difference-in-differences were similarly null among adolescent purchases and purchases made for children (Table 3). Results with continuous underestimation of calories were consistent with those using the binary measure (Additional file 1: Table S3). In adults, as expected, calorie labeling was associated with a substantial increase in predicted probability of noticing calorie information on McDonald’s menus compared to control restaurant menus (β_interaction_ = 28, 95% CI: 21, 36). These results were similar among adolescents, but were weaker among parents/guardians of children (β_interaction_ = 13, 95% CI: − 7, 31). Results for both underestimation of calories and noticing of calorie information on menus were similar when adjusting for additional covariates, though we could not run these analyses in children due to small sample sizes (Additional file 1: Table S4). We found that 28% of adults, 15% of adolescents, and 24% of parents/guardians of children who noticed labels said that they used the label to decide what to purchase. Among all participants in McDonald’s in the post-labeling period, 13% of adults, 6% of adolescents, and 8% of children noticed and used the labels.

**Table 3:** Multivariable-Adjusted Changes (95% CI) in Calorie Underestimation and Noticing of Menu Calorie Information by Adults, Adolescents, and Children After Calorie Labeling in McDonald’s Restaurants Compared to Other Fast-Food Restaurants

	Underestimated Calories Purchased								
	McDonald’s				Other Chains				Difference-in-differences^a^
	Pre	Post	Change (crude)	Change (adjusted)^a^	Pre	Post	Change (crude)	Change (adjusted)^a^	
Adults	63%	64%	1% (−6, 8%)	1% (− 6, 8%)	69%	68%	0% (−5, 4%)	−1% (− 5, 4%)	1% (− 7, 9%)
Adolescents^b,c^	78%	71%	−6% (− 12, 0%)	−5% (− 12, 1%)	76%	71%	− 5% (− 10, − 1%)	− 5% (− 10, − 1%)	−1% (−9, 8%)
Children^c^	68%	67%	−1% (− 17, 14%)	1% (− 16, 17%)	70%	69%	0% (− 14, 13%)	−2% (− 17, 12%)	3% (−18, 24%)
	Noticed Calorie Information								
	McDonald’s				Other Chains				Difference-in-differences^a^
	Pre	Post	Change (crude)	Change (adjusted)^a^	Pre	Post	Change (crude)	Change (adjusted)^a^	
Adults	15%	45%	30% (24, 36%)	30% (24, 37%)	25%	26%	2% (−2, 6%)	2% (−2, 6%)	28% (21, 36%)
Adolescents^b,c^	11%	38%	27% (21, 32%)	26% (20, 32%)	18%	19%	1% (− 4, 5%)	1% (−3, 5%)	25% (18, 32%)
Children^c^	14%	36%	21% (7, 35%)	22% (7, 37%)	18%	25%	7% (−3, 18%)	9% (−2, 21%)	13% (− 7, 31%)

^a^Changes in the predicted probability of the outcome of interest are adjusted for age (years, continuous), sex (female [ref], male), race/ethnicity (white [ref], black, Asian, Hispanic, other), BMI (kg/m^2^, continuous), city (Boston [ref], Hartford, Providence, Springfield), and restaurant chain (Burger King [ref], KFC, Subway, Wendy’s). Restaurant location was included as a random effect

^b^Control restaurants included Burger King (ref), Wendy’s, Subway, and Dunkin’ Donuts

^c^Adjusted for BMI-for-age-and-sex z score (continuous) instead of BMI

When repeating our primary analysis stratified by participant characteristics (Table 4), we observed fewer calories purchased by adolescents with obesity in McDonald’s compared to control restaurants (β_interaction_ = − 246 cal, 95% CI: -500, 9). Calorie labeling was also associated with reduced calories purchased by Black (β_interaction_ = − 128 cal, 95% CI: − 237, − 19) and White adolescents (β_interaction_ = − 141 cal, 95% CI: − 291, 10), but not among Hispanics or in either sex. We did not observe associations of calorie labeling by these characteristics in adults.

**Table 4:** Multivariable-Adjusted Difference-in-Differences (95% CI) for Calories Purchased in Adults and Adolescents by Participant Characteristics

Characteristic	Adults^a^		Adolescents^b^	
	N	Difference-in-differences	N	Difference-in-differences
Sex				
Male	1707	−19 (− 122, 85)	1121	−81 (− 203, 41)
Female	1264	−12 (− 130, 106)	1043	− 12 (− 115, 92)
Obesity Status^c^				
Obesity	889	−112 (− 282, 56)	284	−246 (− 500, 9)
No obesity	2086	19 (− 67, 105)	1880	−30 (− 112, 52)
Race/ethnicity				
Black	906	−33 (− 151, 84)	747	−128 (−237, − 19)
Hispanic	566	−63 (− 330, 203)	577	41 (− 131, 214)
White	1143	−38 (−177, 100)	427	−141 (−291, 10)

^a^Adjusted for age (years, continuous), sex (female [ref], male), race/ethnicity (white [ref], black, Asian, Hispanic, other), BMI (kg/m^2^, continuous), city (Boston [ref], Hartford, Providence, Springfield), and restaurant chain (Burger King [ref], KFC, Subway, Wendy’s). Restaurant location was included as a random effect

^b^Adjusted for same variables as in adults, except BMI-for-sex-and-age z score (continuous) was used instead of BMI, and control restaurants included Burger King (ref), Wendy’s, Subway, and Dunkin’ Donuts

^c^Obesity defined as BMI ≥30 in adults and BMI-for-age-and-sex z score ≥ 1.645 in adolescents

## Discussion

In this natural experiment of adults, adolescents, and children, we found that McDonald’s voluntary calorie labeling was not associated with large overall changes in calorie content of meals purchased compared to unlabeled fast-food restaurant meals. Exploratory analyses revealed that calorie labeling was associated with fewer calories purchased among adolescents with obesity as well as Black and White adolescents. Although there were no associations of calorie labeling in our primary analyses, adult purchases and purchases made for children in both intervention and control groups had fewer calories over time. Moreover, although there was a substantially higher predicted probability of noticing calorie information in McDonald’s after labeling compared to control restaurants among adults and adolescents, calorie labeling was not associated with improvements in estimation of calories purchased.

Our main results are consistent with most previous studies conducted in fast-food restaurants using a natural experiment design [12–17, 20], which have generally found that calorie labeling is associated with noticing calorie information but not with calories purchased. Many of these studies sampled participants from the same chains as in the present study, using data collected in cities that had implemented calorie labeling (i.e. New York City, Philadelphia, and Seattle) compared to control cities that had not. Only two of these studies investigated this association in children and adolescents specifically, and both did not observe any differences in calories purchased after labeling [14, 17]. Although this suggests limited ability of calorie labeling to reduce calories purchased in fast-food settings, one exception is a study conducted by Bollinger et al., which found a significant 15-cal decrease in Starbucks purchases in New York City after labeling (compared to Starbucks locations in Boston and Philadelphia where labeling was not yet implemented) [7]. This study used > 100 million restaurant transactions, allowing them to detect very small differences in calories purchased. No other study to date, including the present study, has been large enough to detect such small differences in calories purchased. However, microsimulation studies have shown that even small reductions (e.g. 8–11 cal/day) could prevent hundreds of thousands of cases of obesity and cardiometabolic disease [25, 26]. Thus, the lack of an association in the present study and in past small studies can be used only as evidence against a moderate or large effect of labeling on calories purchased. They should not be used to rule out a small but meaningful effect of labeling because these studies have generally not been powered to detect such effects. The statistically non-significant reduction of 49 cal among adolescents dining at McDonald’s compared to other chains, pre vs. post, provides further evidence that a small reduction cannot be ruled out in this study.

Although our study included a greater number of child and adolescent participants than previous studies [14, 17], we could not completely separate potential parent and guardian influence over choices. Our sampling of adolescents in the early afternoon in restaurants within one mile of a high school may have increased our chances of enrolling adolescents unaccompanied by adults. However, we did not exclude adolescents who were accompanied by an adult. This is also true for children in our sample, who were all accompanied by an adult. In a study of parent-child dyads by Tandon et al., most parents (~ 70%) reported that the child alone selected the child’s meal [17], whereas Elbel et al. reported that only 31% of children decided on their own what they ate [14]. Purchases made for children in our study, therefore, likely represent some combination of child and adult preferences. However, Elbel et al. also reported no association between parental involvement and fast-food calorie consumption; it is unclear whether parent/guardian influence would have an effect on changes in calorie purchases made after labeling.

Although calorie labeling was not associated with calories purchased in our study overall, we observed a decrease in calories purchased among adolescents with obesity and Black and White adolescents in exploratory analyses. Previous studies have found that individuals with overweight or obesity are more likely to notice or use calorie labels than individuals with underweight or normal weight [27–29]; these studies did not examine calorie purchases specifically. Studies examining calorie label awareness or use by race/ethnicity have been inconsistent, with some finding greater benefits of labeling in White populations and others finding greater benefits in non-White populations [29–31]. Our exploratory analyses do not clarify this question because they suggest similar reductions in calories purchased in Black and White groups. Because we found no associations of calorie labeling with calories purchased among all adolescents, these analyses suggest that this null association may have been driven by participants in the Hispanic, Asian, and Other race/ethnicity groups (the latter two of which we did not conduct subgroup analyses in due to small sample sizes). To our knowledge, this has not been reported previously, though the finding in Black adolescents may be worth further investigation given the higher risk of chronic disease in Black populations [32–34]. Given our lack of a priori hypotheses about the subgroups and the relatively small number of adolescents in each, these results may also be due to chance.

Enhancing consumers’ knowledge of the calorie content of restaurant meals is one mechanism through which calorie labeling aims to reduce calories purchased. This is important considering that consumers frequently underestimate calories in restaurant meals [14, 38, 39]. Indeed, in the present study, more than 60% of participants in the pre-labeling period underestimated calories purchased across age groups, with mean underestimated calories ranging from 111 (adults in McDonald’s) to 260 (adolescents in control chains). However, we did not find any differences in estimation of calories purchased after labeling implementation. This may be a sign that calorie labels are an insufficient means of communicating nutrition information, further supported by the fact that fewer than 50% of McDonald’s customers noticed calorie information after labeling. Other studies have observed similar findings [15, 16, 29]. Although this suggests the need for enhanced promotion of calorie labels, a potentially bigger problem is that even among the minority of customers who noticed the labels, few (15–28% depending on age) used them to inform their meal choices. This likely explains why we observed no differences in calories purchased when restricting our sample to McDonald’s customers who reported noticing labels after implementation (though this analysis might have introduced other biases [40]).

These results together imply that promoting labels or increasing their visibility might not be enough to reduce overall calorie purchases in fast-food settings. More widespread rollout of calorie labeling across the U.S. might increase the salience of this information over time. However, many fast-food consumers may just not find this information helpful; about half of participants responded that calories were “not at all” important in their meal selection. Other types of health communication, such as traffic light labels, may better communicate the importance of energy balance for health [41], and labels might be more effective in other settings like full-service chain restaurants [42, 43] or cafeterias [10, 11, 44, 45].

The natural experiment design we used, which accounted for secular trends in calorie purchases and included a control group with similar menu offerings and clientele as our intervention group, make our results more robust to unmeasured confounding [46]. This allowed us to draw more valid inferences. Other strengths include our repeated sampling in the same restaurant locations over a five-year period spanning labeling implementation, and a racially and ethnically diverse population.

There are also several limitations to this study. First, although difference-in-differences analyses are generally robust to unmeasured confounding, it is possible that there were some characteristics that changed between groups over time that we did not measure. This could confound our results if these characteristics are related to calorie intake. However, we measured the major characteristics that we felt were most likely to be confounders and adjusted for these variables even though their distributions seemed to differ only slightly between groups over time. Second, even though this study is, to our knowledge, the largest to date of adolescents and children (and one of the largest of adults), we had low power to detect small differences in calories purchased after labeling implementation. Third, although our results are generalizable to individuals of different racial/ethnic groups, participation rates were < 50% across samples, suggesting we may have enrolled a select population among eligible individuals, such as people with lower incomes (i.e. those more receptive to a $2 incentive) and those who primarily do not use drive-thrus (from whom we did not collect data), where calorie labels may be visualized differently than in restaurants. Our results therefore may not be generalizable to all fast-food consumers in the U.S., though our sampling of participants from top-selling chains may enhance this generalizability. Given some differences in menu offerings of these restaurants in different countries, and differences in the demographic composition and health literacy of their clientele, it is unclear how generalizable these results are to customers outside of the U.S. Fourth, individuals’ purchases may have been influenced by participation in the study. However, we expect that any change in purchases due to participation in this study would be similar in McDonald’s and control restaurants and would not greatly affect our main results. When approaching potential participants (prior to their orders), we provided only minimal information about the study, stating that the study was about “food and drink choices at fast-food restaurants.” Fifth, we only assessed purchased, not consumed food. If calorie labeling is associated with reduced intake, but not purchases, of restaurant meals, our results could be underestimated. Last, labeling may have caused some people to forego dining at McDonald’s, but we were not able to capture this information due to the repeated cross-sectional nature of our study. People who decided not to purchase food from McDonald’s due to labeling might have consumed more or fewer calories for that meal at a different location than at McDonald’s. A previous study in Philadelphia found that individuals did not change their frequency of fast-food visits after labeling [15], but more information on substitution is needed to understand the effect of labeling on overall diet quality.

## Conclusions

In summary, we did not observe large differences in actual or estimated calorie content of meals purchased in McDonald’s restaurants after calorie labeling when compared to control restaurants in adults, adolescents, or children. These findings may be partially explained by the still incomplete recognition of calories, even when labeling is present. However, it is also possible that any true effect of calorie labeling is smaller than we could detect with our sample sizes. Although there were no associations of calorie labeling on purchases, the calories purchased by adults and adolescents declined over time, suggesting this might be a secular trend that is driven by myriad factors, one of which could be labeling or the anticipation of labeling.

The recent nationwide implementation of the calorie labeling law offers future opportunities to investigate these effects in larger studies, particularly in full-service restaurants, where the effect of calorie labeling on diet quality may be stronger [42]. Because of the now widespread implementation of labeling, chains might offer lower calorie options, decreasing calorie content by default [47], which should also be examined further. The law additionally requires labeling of prepared foods in supermarkets; no studies have investigated labeling in these settings [20]. Other areas for future research include evaluation of the effects of calorie labeling on overall diet quality, including nutrient and food group composition of purchases [48]. Lastly, calorie labeling in the presence of other policies for obesity prevention (e.g. beverage taxes) should be investigated because there may be synergistic effects that have a positive impact on diet.

## Supplementary information

**Additional file 1: Table S1.** Multivariable-Adjusted Changes (95% CI) in Calories Purchased by Adults, Adolescents, and Children After Calorie Labeling in McDonald’s Restaurants Compared to Other Fast Food Restaurants After Adjusting for Additional Participant Characteristics. **Table S2.** Multivariable-Adjusted Changes (95% CI) in Calories Purchased After Calorie Labeling in McDonald’s Restaurants Compared to Other Fast Food Restaurants After Excluding McDonald’s Customers Who Did Not Report Seeing Calories on Menus in the Post Period. **Table S3.** Multivariable-Adjusted Changes (95% CI) in Underestimation of Calories Purchased (Continuous) by Adults, Adolescents, and Children After Calorie Labeling in McDonald’s Restaurants Compared to Other Fast Food Restaurants. **Table S4.** Multivariable-Adjusted Percent Changes (95% CI) in Calorie Underestimation and Noticing of Menu Calorie Information by Adults and Adolescents After Calorie Labeling in McDonald’s Restaurants Compared to Other Fast Food Restaurants After Adjusting for Additional Participant Characteristics.

## Data Availability

Data are available upon request as long as proper data use agreements are executed and approved by the Harvard Pilgrim Health Care Office of Sponsored Programs and Institutional Review Board.
