# Supplementary material for: Evaluation of the impact of calorie labeling on McDonald’s restaurant menus: a natural experiment
Source: Int J Behav Nutr Phys Act. 2019 Nov 4;16:99. doi: 10.1186/s12966-019-0865-7 (PMC6829981; doi:10.1186/s12966-019-0865-7)
Supplement: Supplementary file 1 — Additional file 1: Table S1. Multivariable-Adjusted Changes (95% CI) in Calories Purchased by Adults, Adolescents, and Children After Calorie Labeling in McDonald’s Restaurants Compared to Other Fast Food Restaurants After Adjusting for Additional Participant Characteristics. Table S2. Multivariable-Adjusted Changes (95% CI) in Calories Purchased After Calorie Labeling in McDonald’s Restaurants Compared to Other Fast Food Restaurants After Excluding McDonald’s Customers Who Did Not Report Seeing Calories on Menus in the Post Period. Table S3. Multivariable-Adjusted Changes (95% CI) in Underestimation of Calories Purchased (Continuous) by Adults, Adolescents, and Children After Calorie Labeling in McDonald’s Restaurants Compared to Other Fast Food Restaurants. Table S4. Multivariable-Adjusted Percent Changes (95% CI) in Calorie Underestimation and Noticing of Menu Calorie Information by Adults and Adolescents After Calorie Labeling in McDonald’s Restaurants Compared to Other Fast Food Restaurants After Adjusting for Additional Participant Characteristics. [file 12966_2019_865_MOESM1_ESM.docx]

| **Supplemental Table 1. Multivariable-Adjusted Changes (95% CI) in Calories Purchased by Adults, Adolescents, and Children After Calorie Labeling in McDonald's Restaurants Compared to Other Fast Food Restaurants After Adjusting for Additional Participant Characteristics** | | | | | | | | | |
| --- | --- | --- | --- | --- | --- | --- | --- | --- | --- |
|  | **Calories Purchased** | | | | | | | | |
|  | **McDonald's** | | |  | **Other Chains** | | |  | **Difference-in-differences^2^** |
|  | **Pre^1^** | **Post^1^** | **Change (adjusted)^2^** |  | **Pre^1^** | **Post^1^** | **Change (adjusted)^2^** |  |  |
| **Adults** | 718 (474) | 630 (425) | -67 (-139, 4) |  | 888 (455) | 816 (468) | -54 (-108, 0) |  | -18 (-109, 73) |
| **Adolescents^3,4^** | 759 (488) | 709 (408) | -38 (-96, 19) |  | 756 (427) | 739 (459) | -6 (-52, 41) |  | -45 (-125, 35) |
| **Children^4^** | 719 (369) | 585 (280) | -177 (-258, -95) |  | 766 (345) | 585 (345) | -161 (-259, -62) |  | -3 (-131, 126) |
| ^1^Mean (SD) presented | | | | | | | | | |
| ^2^Adjusted for age (years, continuous), sex (female [ref], male), race/ethnicity (white [ref], black, Asian, Hispanic, other), BMI (kg/m^2^, continuous), city (Boston [ref], Hartford, Providence, Springfield), restaurant chain (Burger King [ref], KFC, Subway, Wendy's), importance of price (none [ref], somewhat, very), importance of taste (none [ref], somewhat, very), importance of convenience (none [ref], somewhat, very), importance of calories (none [ref], somewhat, very), and estimated calorie requirement (correct [ref], underestimated, overestimated). Restaurant location was included as a random effect. | | | | | | | | | |
| ^3^Control restaurants included Burger King (ref), Wendy's, Subway, and Dunkin' Donuts | | | | | | | | | |
| ^4^Adjusted for BMI-for-age-and-sex z score (continuous) instead of BMI | | | | | | | | | |

| **Supplemental Table 2. Multivariable-Adjusted Changes (95% CI) in Calories Purchased After Calorie Labeling in McDonald's Restaurants Compared to Other Fast Food Restaurants After Excluding McDonald's Customers Who Did Not Report Seeing Calories on Menus in the Post Period** | | | | | | | | | |
| --- | --- | --- | --- | --- | --- | --- | --- | --- | --- |
|  | **McDonald's** | | |  | **Other Chains** | | |  | **Difference-in-differences^2^** |
|  | **Pre^1^** | **Post^1^** | **Change^2^** |  | **Pre^1^** | **Post^1^** | **Change^2^** |  |  |
| **Adults** | 718 (474) | 624 (412) | -87 (-189, 16) |  | 888 (455) | 816 (468) | -60 (-115, -5) |  | -28 (-145, 90) |
| **Adolescents^3,4^** | 759 (488) | 714 (429) | -38 (-133, 58) |  | 756 (427) | 739 (459) | 2 (-48, 50) |  | -49 (-151, 53) |
| **Children^4^** | 719 (369) | 561 (280) | -132 (-225, -40) |  | 766 (345) | 585 (345) | -159 (-257, -61) |  | 28 (-113, 168) |
| ^1^Mean (SD) presented | | | | | | | | | |
| ^2^Adjusted for age (years, continuous), sex (female [ref], male), race/ethnicity (white [ref], black, Asian, Hispanic, other), BMI (kg/m^2^, continuous), city (Boston [ref], Hartford, Providence, Springfield), and restaurant chain (Burger King [ref], KFC, Subway, Wendy's). Restaurant location was included as a random effect. | | | | | | | | | |
| ^3^Control restaurants included Burger King (ref), Wendy's, Subway, and Dunkin' Donuts | | | | | | | | | |
| ^4^Adjusted for BMI-for-age-and-sex-z score (continuous) instead of BMI | | | | | | | | | |

| **Supplemental Table 3. Multivariable-Adjusted Changes (95% CI) in Underestimation of Calories Purchased (Continuous) by Adults, Adolescents, and Children After Calorie Labeling in McDonald's Restaurants Compared to Other Fast Food Restaurants** | | | | | | | | | |  |
| --- | --- | --- | --- | --- | --- | --- | --- | --- | --- | --- |
|  | **Calories Underestimated** | | | | | | | | |  |
|  | **McDonald's** | | |  | **Other Chains** | | |  | **Difference-in-differences^2^** | |
|  | **Pre^1^** | **Post^1^** | **Change^2^** |  | **Pre^1^** | **Post^1^** | **Change^2^** |  |  | |
| **Adults** | 119 (608) | 110 (507) | -6 (-87, 75) |  | 196 (639) | 170 (631) | -27 (-86, 31) |  | 25 (-73, 123) | |
| **Adolescents^3,4^** | 246 (581) | 144 (524) | -93 (-198, 12) |  | 261 (530) | 218 (575) | -29 (-106, 47) |  | -71 (-201, 59) | |
| **Children^4^** | 162 (597) | 113 (489) | -31 (-195, 133) |  | 160 (610) | 88 (476) | -77 (-265, 111) |  | 51 (-190, 293) | |
| ^1^Mean (SD) presented | | | | | | | | | |  |
| ^2^Adjusted for age (years, continuous), sex (female [ref], male), race/ethnicity (white [ref], black, Asian, Hispanic, other), BMI (kg/m^2^, continuous), city (Boston [ref], Hartford, Providence, Springfield), and restaurant chain (Burger King [ref], KFC, Subway, Wendy's). Restaurant location was included as a random effect. | | | | | | | | | |  |
| ^3^Control restaurants included Burger King (ref), Wendy's, Subway, and Dunkin' Donuts | | | | | | | | | |  |
| ^4^Adjusted for BMI-for-age-and-sex z score (continuous) instead of BMI | | | | | | | | | |  |

| **Supplemental Table 4. Multivariable-Adjusted Percent Changes (95% CI) in Calorie Underestimation and Noticing of Menu Calorie Information by Adults and Adolescents After Calorie Labeling in McDonald's Restaurants Compared to Other Fast Food Restaurants After Adjusting for Additional Participant Characteristics** | | | | | | | | | |
| --- | --- | --- | --- | --- | --- | --- | --- | --- | --- |
|  | **Underestimated Calories Purchased** | | | | | | | | |
|  | **McDonald's** | | |  | **Other Chains** | | |  | **Difference-in-differences^1^** |
|  | **Pre** | **Post** | **Change^1^** |  | **Pre** | **Post** | **Change^1^** |  |  |
| **Adults** | 63% | 64% | 1% (-6%, 8%) |  | 69% | 68% | -1% (-5%, 4%) |  | 2% (-7%, 10%) |
| **Adolescents^2^** | 78% | 71% | -4% (-11%, 2%) |  | 76% | 71% | -5% (-10%, -1%) |  | 1% (-7%, 9%) |
|  | **Noticed Calorie Information** | | | | | | | | |
|  | **McDonald's** | | |  | **Other Chains** | | |  | **Difference-in-differences^1^** |
|  | **Pre** | **Post** | **Change^1^** |  | **Pre** | **Post** | **Change^1^** |  |  |
| **Adults** | 15% | 45% | 32% (26%, 38%) |  | 25% | 26% | 1% (-3%, 5%) |  | 30% (23%, 38%) |
| **Adolescents^2^** | 11% | 38% | 26% (20%, 32%) |  | 18% | 19% | 2% (-3%, 6%) |  | 25% (18%, 32%) |
| ^1^Changes in the predicted probability of the outcome of interest are adjusted for age (years, continuous), sex (female [ref], male), race/ethnicity (white [ref], black, Asian, Hispanic, other), BMI (kg/m^2^, continuous), city (Boston [ref], Hartford, Providence, Springfield), restaurant chain (Burger King [ref], KFC, Subway, Wendy's), importance of price (none [ref], somewhat, very), importance of taste (none [ref], somewhat, very), importance of convenience (none [ref], somewhat, very), importance of calories (none [ref], somewhat, very), and estimated calorie requirement (correct [ref], underestimated, overestimated). Restaurant location was included as a random effect. | | | | | | | | | |
| ^2^Control restaurants included Burger King (ref), Wendy's, Subway, and Dunkin' Donuts. We adjusted for BMI-for-age-and-sex z-score (continuous) instead of BMI | | | | | | | | | |
